# Buffered delivery of phosphate to Arabidopsis alters responses to low phosphate

**DOI:** 10.1093/jxb/erx454

**Published:** 2018-01-03

**Authors:** Meredith T Hanlon, Swayamjit Ray, Patompong Saengwilai, Dawn Luthe, Jonathan P Lynch, Kathleen M Brown

**Affiliations:** 1 Department of Plant Science and Intercollege Graduate Degree Program in Plant Biology, Pennsylvania State University, University Park, PA, USA; 2 Department of Entomology, Pennsylvania State University, University Park, PA, USA; 3 Department of Biology, Faculty of Science, Mahidol University, Rama VI Road, Rachadhavi, Bangkok, Thailand

**Keywords:** Arabidopsis, gel media, lateral roots, low phosphate, root growth, root hairs

## Abstract

Arabidopsis has been reported to respond to phosphate (Pi) stress by arresting primary root growth and increasing lateral root branching. We developed a system to buffer Pi availability to Arabidopsis in gel media systems by charging activated aluminum oxide particles with low and sufficient concentrations of Pi, based on previous work in horticultural and sand culture systems. This system more closely mimics soil chemistry and results in different growth and transcriptional responses to Pi stress compared with plants grown in standard gel media. Low Pi availability in buffered medium results in reduced root branching and preferential investment of resources in axial root growth. Root hair length and density, known responses to Pi stress, increase in low-buffered Pi medium. Plants grown under buffered Pi conditions have different gene expression profiles of canonical Pi stress response genes as compared with their unbuffered counterparts. The system also eliminates known complications with iron (Fe) nutrition. The growth responses of Arabidopsis supplied with buffered Pi indicate that the widely accepted low-Pi phenotype is an artifact of the standard gel-based growth system. Buffering Pi availability through the method presented here will improve the utility and accuracy of gel studies by more closely approximating soil conditions.

## Teaser

The responses of Arabidopsis to low-phosphate conditions are different from previously described when phosphate is supplied through solid-phase buffered delivery that approximates soil chemistry.

## Introduction

Global food production must increase by 70% by 2050 (FAO, 2009). Pressures due to climate change, and the increasing price of fertilizer (Elser *et al.*, 2014) will only exacerbate this challenge. Over half of global agricultural land is low in available phosphorus (P), and this is especially true in tropical regions such as sub-Saharan Africa, where the greatest population increases are expected to occur (Lynch, 2011). Understanding plant adaptation to low P availability is of inherent interest in plant biology, while also being strategically important for the development of more resilient, productive agricultural systems (Vance *et al.*, 2003).

P is an essential macronutrient, required in large quantities throughout plant growth and development. In soil, P is available to plants as soluble inorganic phosphate (Pi). Plants have developed various mechanisms to obtain and efficiently use Pi to cope with limited environmental availability, and these adaptations have long been studied in a variety of species. Ideotypes for maximum Pi uptake have been developed through modeling and empirical studies (Ge *et al.*, 2000; Liao *et al.*, 2001; Lynch and Brown, 2001, 2012; Ho *et al.*, 2004; Wang *et al.*, 2010*b*; Fang *et al.*, 2011; Lynch, 2011; Richardson *et al.*, 2011; Heppell *et al.*, 2015). Phosphate is usually found in shallow soil layers (Heppell *et al.*, 2016); therefore, shallow root growth angles and increased root growth in shallow soil layers that promote topsoil foraging are advantageous for Pi acquisition (Lynch, 2011; Miguel *et al.*, 2013; White *et al.*, 2013). Increased root hair density (RHD) also contributes to enhanced Pi acquisition at minimal cost to the plant (Gahoonia and Nielsen, 1997, 2004; Bates and Lynch, 2000, 2001; Ma *et al.*, 2001; Brown *et al.*, 2013). Reducing the carbon cost of roots through decreased living cortical area or reduced respiration improves maize and common bean growth under Pi-limiting conditions (Nielsen *et al.*, 2001; Postma and Lynch, 2011; Lynch and Wojciechowski, 2015). Increasing arbuscular mycorrhizal symbioses and exploiting microbial P solubilization can enhance plant P acquisition (Smith and Read, 2010; Richardson and Simpson, 2011).

In fertile soil, P concentrations are 100- to 1000-fold lower than concentrations found in typical Arabidopsis growth media, with soil levels ranging from 1 μM to 10 μM P (Bieleski, 1973). P availability and concentration are highly dynamic. P is bound and released from soil constituents into the soil solution to maintain a small, soluble pool of plant-available Pi, which makes up 0.01% of total soil P (Kruse *et al.*, 2015). Pi is bound by iron and aluminum oxides in acidic soils, and calcium in alkaline soils, making the availability of P to plants dependent on the concentrations of these elements. Pi availability is also dependent on the soil pH, and the surface area and structure of the soil particles (Holford, 1997). These factors determine the buffering capacity or sorptivity of the soil, and small changes in the buffering capacity value can cause variations in plant P uptake by up to 50% (Heppell *et al.*, 2016). P is constantly being precipitated and solubilized, and adsorbed and desorbed (Heuer *et al.*, 2017), resulting in a highly spatially and temporally dynamic system. In the rhizosphere, sharp gradients of Pi are formed as uptake of Pi at the root surface decreases the local Pi concentration. The rate of replacement is limited by the physical and chemical characteristics of the specific soil (Hinsinger, 2001). P bound to solid phases in the soil is maintained in equilibrium with the available Pi in the soil solution, which moves by diffusion (Pierzynski and McDowell,2005). This dynamic process allows plant tissues to accumulate levels of P that are at least 1000 times greater than the soluble soil concentration measured at a single moment in time (Raghothama, 1999).

Arabidopsis has served as an important model for understanding the molecular basis of Pi stress responses and adaptations. The large majority of these studies have employed gel-based media with unrealistically high nutrient availability. Gel systems allow direct observation of rapid and uniform growth, but differ greatly from natural soil environments. Here, we present a modified gel-based system that allows for rapid growth and easy observation of root development while more closely resembling natural Pi regimes in soil, since the Pi is delivered through a buffered mechanism. In classic gel systems, Pi is freely available; our system effectively buffers the delivery of Pi by employing aluminum oxide (Al_2_O_3_) surface chemistry, as occurs in natural soil (Elliott *et al.*, 1983; Elliott, 1989; Lynch *et al.*, 1990; Oh *et al.*, 2016).

Concerns over the dynamics of solution culture versus soil for plant growth have been raised previously (Mackay and Barber, 1984). Hundreds of studies have used gel systems to study Pi responses in Arabidopsis, with new results on individual genes, pathways, and interactions with other nutrients reported regularly. Initial work describing the effects of Pi stress on the Arabidopsis root system indicated that Pi stress results in a shorter primary root with increased lateral root branching (Williamson *et al.*, 2001). Subsequent research demonstrated that auxin controls this determinate phenotype of the primary root (López-Bucio *et al.*, 2002; Nacry *et al.*, 2005; Sánchez-Calderón *et al.*, 2005). Some of the most recent papers published on Pi stress further explore this response and have identified mutants that lack a short-root phenotype (Karthikeyan *et al.*, 2014), showed how the Pi response is related to other nutrient stresses (Kellermeier *et al.*, 2014), and identified large gene networks involved in the Pi response (Karthikeyan *et al.*, 2014; Kellermeier *et al.*, 2014; Salazar-Henao and Schmidt, 2016; Sun *et al.*, 2016; Mora-Macías *et al.*, 2017). Recent reviews discuss the roles of individual genes and suggest that further studies of these genes will help with improving crop Pi acquisition (Bouain *et al.*, 2016; Gu *et al.*, 2016; Heuer *et al.*, 2017).

Unlike what is commonly reported in Arabidopsis, low-Pi conditions do not favor prolific lateral root branching in maize, common bean, or rice, but instead result in increased axial root elongation compared with lateral root elongation (Borch *et al.*, 1999; Mollier and Pellerin, 1999; Vejchasarn *et al.*, 2016). In typical low-P soils, plants must increase soil, especially topsoil, exploration (Lynch, 2011). A branching pattern that favors increased soil exploration rather than root proliferation into soil domains where P is scarce is a more logical strategy. Increasing growth at minimal cost, by growing thinner roots, forming aerenchyma, or relying on metabolically cheap root hairs for Pi uptake, are strategies that lead to increased soil exploration (Lynch, 2011). Soil-grown Arabidopsis plants have decreased total shoot and root biomass and increased specific root length in low Pi, indicating that roots are increasing the soil volume explored, not investing energy in local growth (Nord and Lynch, 2008), and lateral root branching does not increase in relation to Pi deprivation (Linkohr *et al.*, 2002). The arrest of the primary root in gel media is due to an overabundance of available iron (Fe) in low-Pi media (Ward *et al.*, 2008; Bournier *et al.*, 2013), and is not solely due to Pi limitation. This phenotype may have arisen due to the gel growth system that eliminates soil chemistry and has minimal buffering capacity.

Here, we describe a system to buffer P delivery in a gel-based Arabidopsis growth system. To mimic soil P dynamics more closely, Pi was bound to activated Al_2_O_3_ particles (Lynch *et al.*, 1990; Gourley *et al.*, 1993). This system has been used in greenhouse and horticultural studies (Borch *et al.*, 1998; Brown *et al.*, 1999; Tanaka *et al.*, 2006; Vejchasarn *et al.*, 2016), but, until now, has not been adapted for plant growth in gel-based media. In this system, Al_2_O_3_ particles are adsorbed with Pi, resulting in a solid-phase buffered P system that supplies realistic low P regimes to the plant (Lynch *et al.*, 1990; Borch *et al.*, 1999; Oh *et al.*, 2016). An equilibrium concentration of Pi is maintained in solution, allowing for a diffusion-limited Pi supply, similar to soil (Elliott, 1989; Lynch *et al.*, 1990). The non-soluble Al_2_O_3_ particles do not expose the plants to available aluminum (Lynch *et al.*, 1990). Buffered P delivery results in smaller plants with reduced lateral root branching density, long root hairs, and altered expression of canonical Pi stress response genes when compared with plants grown on media with a low concentration of unbuffered P. The buffered, aluminum–Pi (Al-P) system allows for consistent, controlled Pi regimes for the plants. In traditional unbuffered gel systems, plant Pi uptake can lead to depletion of media Pi that results in plant Pi starvation, not deprivation. Our system results in root growth responses that are similar to those of low-Pi soil-grown plants. Here, we show that a solid-phase buffered P system for traditional, gel-based Arabidopsis growth results in a realistic and sustained P stress throughout plant growth.

## Materials and methods

### Buffered phosphorus preparation

Compalox raw alumina (AN/V-801; Albemarle/Martenswerk, Germany) was charged with P at various concentrations following a modified protocol based on earlier work (Lynch *et al.*, 1990). Briefly, the alumina was sieved to a size from 400 µm to 650 µm in diameter, rinsed with Millipore-filtered water for 15 min, acidified by adding 0.0406 N HCl, and shaken for another 15 min. The alumina was rinsed with water until the pH of the rinse reached 4.3 and was then loaded with an appropriate concentration of P in the form of KH_2_PO_4_ in 0.01 N NaCl by shaking for 2 h; for low-Pi alumina, a concentration of 80 mM KH_2_PO_4_ was used, and for medium-Pi alumina, a concentration of 400 mM KH_2_PO_4_ was used. Three additional rinses with Millipore-filtered water were completed before the loaded alumina was dried and analyzed for Pi desorption using the methods of Murphy and Riley (1962).

### Growth media preparation

Media were prepared using modified half-strength Epstein solution with adjustments to micronutrients to balance any effects of the application of the alumina particles (see Supplementary Fig. S1 at *JXB* online). Media were adjusted to a pH of 5.7 and solidified using 0.8% agar (PhytoTechnology Laboratories, Shawnee Mission, KS, USA). Media for both unbuffered and buffered P delivery contained 3 mM KNO_3_, 2 mM Ca(NO_3_)_2_·4H_2_O, 0.5 mM MgSO_4_·7H_2_O, 49.95 µM KCl, 2 µM MnSO_2_·2H_2_O, 0.5 µM CuSO_4_·5H_2_O, and 50 µM Fe-NaDTPA (Dissolvine, D-FE-11, AkzoNobel, Amsterdam, The Netherlands). Pi concentrations were chosen to represent a range of Pi used to study Pi responses. Unbuffered high-Pi media contained 1 mM NH_4_H_2_PO_4_, unbuffered medium Pi, 50 µM NH_4_H_2_PO_4_, and unbuffered low Pi contained no additional NH_4_H_2_PO_4_. Ions were balanced by the addition of 0.475 mM (NH_4_)_2_SO_4_ to unbuffered medium-Pi solution and 0.5 mM (NH_4_)_2_SO_4_ to unbuffered low-Pi solution and all buffered solutions.

Micronutrients in unbuffered media consisted of 25 µM H_3_BO_3_, 2 µM Zn-Na_2_EDTA, and 0.5 µM (NH_4_)_6_Mo_7_O_24_·4H_2_O. In buffered media, these three micronutrients were adjusted to account for differences due to the addition of alumina to 48.25 µM H_3_BO_3_, 8.9 µM Zn-Na_2_EDTA, and 1.26 µM (NH_4_)_6_Mo_7_O_24_·4H_2_O as previously indicated (Gourley *et al.*, 1993). These adjustments were sufficient to overcome interactions between the aluminum particles and these nutrients as determined by plant elemental content [boron (B) and zinc (Zn)] or nitrate reductase activity [molybdenum (Mo)] (Supplementary Fig. S1). For low-iron experiments, the Fe-NaDTPA concentration was reduced to 2.5 µM. All media were supplemented with 0.125 µM MES, 0.025 µM myo-inositol, and 1% (w/v) sucrose.

Alumina particles were added to buffered media following separate autoclaving of the two entities. Particles were placed in folded filter paper and sealed prior to autoclaving. Particles (1%, w/v) were evenly distributed directly from the filter paper packet over the top of the media once they had solidified in the plates.

For soil-grown plant experiments, plants were grown in one of three mixtures: 100% low-P [3 µM available P (Olsen *et al.*, 1954)] field soil [Hagerstown-Opequon, fine clayey, mixed, mesic (Typic Hapludalf), autoclaved 6 months before use], sand:vermiculite:low-P buffered alumina [60:39:1 (v/v/v)], or sand:vermiculite:field soil [48:32:28 (v/v/v)]. Plants were fertilized with either high- or low-P half-strength Epstein nutrient solution.

### Plant growth

Seeds (*Arabidopsis thaliana* Col-0) were sterilized in 10% bleach for 5 min and rinsed a minimum of four times with sterile water. Seeds were plated, stratified at 4 °C for 2 d, and moved to 22 °C growth chambers with a long-day (16 h) light cycle and light levels from 105 µmol m^–2^ s^–1^ to 120 µmol m^–2^ s^–1^. Plates were placed vertically to ensure growth along the surface of the agar and exposure to the aluminum particles.

For soil-grown plants, seeds were imbibed in water for 48 h at 4 °C. Seeds were then planted on top of the media and grown in 6.35 cm pots in the same growth chamber as the plates to ensure similar conditions. Plants were harvested after 12 d.

### Data collection and analysis

Plates were scanned using a conventional flatbed scanner with a resolution of 300 dpi 3, 6, 9, and 12 d after germination. Root architecture analysis was completed using the semi-automated tracing program, RootNav (Pound *et al.*, 2013), and measurements were extracted and analyzed using R (http://www.R-project.org). For plant weights, five plants from each plate were pooled into a single group.

Images for measurements of root hair length and density were obtained at 30× magnification on a dissecting microscope equipped with a CCD camera (SMZ-U and NIKON DS-Fi1, Nikon, Tokyo, Japan). Images were taken of the primary root 1 cm from the root tip. Root hair density was measured by counting along the root edge. The lengths of 10 root hairs were measured per plant using ImageJ software (National Institute of Mental Health, Bethesda, MD, USA) and data analyzed in R.

Images for epidermal cell length were obtained by first staining roots with 0.05% toluidine blue and then taking images at 80× magnification on a dissecting microscope equipped with a CCD camera (SMZ-U and NIKON DS-Fi1). Five trichoblasts and five atrichoblasts were measured on each root sample 1 cm from the primary root tip.

Cortical cell counts were obtained from sections sampled 1–2 cm from the root tip and imaged on a Nikon Diaphot inverted microscope equipped with a CCD camera (SMZ-U and NIKON DS-Fi1) using a 10× objective. Primary root segments 3 cm in length (tip included) were embedded in a 3% agar (PhytoTechnology Laboratories) solution to aid with hand sectioning. Sections were briefly stained with 0.05% toluidine blue prior to imaging. Images were analyzed in ImageJ with the ObjectJ (https://sils.fnwi.uva.nl/bcb/objectj/index.html) plugin.

Total plant and gel P concentrations were analyzed using the methods of Murphy and Riley (1962). For buffered treatments, Al_2_O_3_ particles were manually removed prior to gel drying and ashing. Other nutrient contents were determined either via ICP-OES (inductively coupled plasma optical emission spectrometry) for gel or ICP-MS for plant tissue. Gel nutrient analysis was completed following drying (60 °C for 4 d), ashing (243 °C for 8 h), and digestion in 0.1 N HCl. Digests were analyzed on an Agilent 730-ES, axial torch orientation (Agilent Technologies, Santa Clara, CA, USA) following EPA method 6010. Plant nutrient analysis was completed following drying (60 °C for 4 d) and digestion in concentrated, ultra-pure nitric acid (HNO_3_) for 8 h at 80 °C in teflon vessels. The solution was diluted to obtain a final HNO_3_ of 0.45 N. Samples were run on a Thermo Fisher Scientific XSeries 2 both with and without collision cell technology as required (srm 1640a, NIST 1547 as standard). Nitrate reductase activity was determined following published methods (Cheeseman and Tankou, 2005). Protein concentration was determined via a Bradford assay (B6916 Sigma-Aldrich) using BSA as a standard.

Statistical significance was determined using either a one- or two-way ANOVA, with Tukey’s honest significant difference (HSD) tests via the ghlt command in the multcomp package in R. Data were transformed prior to analysis as necessary using either a natural log or box-cox transformation. For all growth experiments, a minimum of 20 plants were analyzed per treatment, and all experiments were repeated at least three times.

### RNA extraction, cDNA synthesis, and quantitative real-time PCR

Roots from five Arabidopsis plants from the same plate were pooled to one tube and treated as one biological replicate for the gene expression studies. Four plates were harvested, and this was repeated three times for the analysis of each gene. RNA was extracted from the harvested tissues with 1 ml of TriZol (Life Technologies, USA) using the manufacturer’s protocol. Genomic DNA contamination was removed from the extracted RNA with 2 U of DNase II (New England Biolabs, USA) for 1 h at 37 °C. The DNase was heat inactivated at 65 °C for 10 min and the RNA was quantified with Nanodrop (Thermofisher Scientific, USA). A total of 500 ng of RNA was reverse transcribed with a cDNA kit (Life Biotechnologies, USA) using 2.5 mM oligo(dT) to make cDNA of only the mRNA. The cDNA was diluted 10 times and quantitative real-time PCR was performed in a 7500 Fast Real Time PCR (Applied Biosystems, USA) with SYBR green as the reporter dye (Roche, USA). Actin7 was used as an internal control to calculate the relative abundance of gene expression using the ΔΔCt method (Livak and Schmittgen, 2001). Primer sequences of the genes for which the relative abundances were measured are included in Supplementary Table S1.

## Results

### Buffered delivery of phosphorus provides a consistent, precise level of Pi to plants

**Fig. 1.**
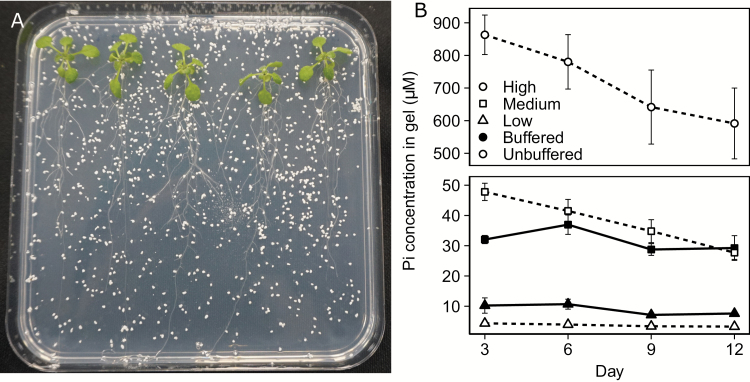
Alumina-buffered phosphate (Al-P) system of Arabidopsis growth. (A) Sample image of 12-day-old Arabidopsis plants grown on the surface of agar with added Al-P particles. (B) Average content (±SE) of Pi in a 50 ml gel plate, *n*=8 for each treatment and time point. ‘Buffered’ refers to use of the Al_2_O_3_ method, whereas ‘unbuffered’ is the standard gel media system.

Concentrations of Pi in the gel of buffered plates (soluble Pi) were 30 µM (medium) and 8 µM (low) throughout the whole plate and stayed nearly constant, decreasing by 16% in low-Pi media and 9% in medium-Pi media, throughout the 12 d duration of the experiment (Fig. 1B, filled circles). Unbuffered plates, on the other hand, had initial Pi concentrations of 900 µM (high), 50 µM (medium), and 6 µM (low), which depleted by 32, 23, and 25% over time, respectively (Fig. 1).

### Plants grown with buffered phosphate require much lower Pi concentrations for sufficient growth

**Fig. 2.**
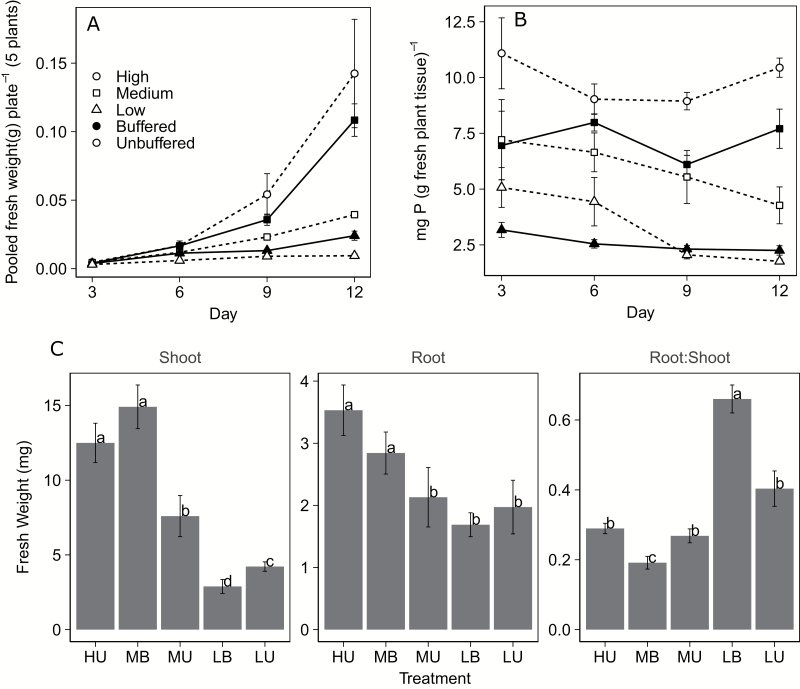
Plant growth and P content on different media. (A) Average pooled fresh weights of all plants (five) on a single plate. Eight plates were collected at each time point (error bars=SE). (B) Plant P content (mg) as determined per gram of fresh plant tissue (*n*=8 per treatment and time point; error bars=SE). (C) Individual plant root and shoot fresh weights and the ratio between the two at 12 DAG (*n*=30 plants per treatment; error bars=SE). Significance determined via Tukey’s HSD, *P*<0.05. Media abbreviations: HU=high Pi, unbuffered; MB=medium Pi, buffered; MU=medium Pi, unbuffered; LB=low Pi, buffered; LU=low Pi, unbuffered.

Plants had different growth rates under different Pi conditions, as shown by measuring the pooled fresh weight of five plants grown together on a single plate (Fig. 2A). By 9 days after germination (DAG), treatment had a highly significant (*F*=42.075, *P*<0.001) effect on plant fresh weight. Plants grown under high, unbuffered Pi (HU) and plants grown under medium, buffered Pi (MB) conditions were significantly larger than all others, a difference that was magnified by 12 DAG. Low, buffered Pi (LB) plants were similar in weight to those grown under medium, unbuffered Pi (MU) conditions, but those grown under LU conditions were significantly smaller (*F*=41.254, *P*<0.05).

Plants grown under HU conditions had the greatest Pi concentration throughout the duration of the experiment (*P*<0.001 at 12 DAG) (Fig. 2B). Plants grown under MB conditions had a steady Pi concentration over time, and had a significantly greater Pi concentration than plants grown under MU conditions and those grown under either LB or LU. Plants grown under LB and LU had Pi concentrations that were significantly less than all other groups, though these two groups were statistically indistinguishable at 12 DAG (Fig. 2B). Pi concentrations in LB and LU plants were significantly less at 12 DAG compared with 3 DAG (*F*=3.2725, *P*<0.05 and *F*=18.45, *P*<0.05 for buffered and unbuffered, respectively). Plants grown under LU conditions displayed the greatest decrease in Pi concentration over time.

Plants grown under P stress tend to allocate more resources to root growth in order to increase foraging and nutrient acquisition (Hawkesford *et al.*, 2012). We assessed this by calculating the root to shoot ratio at 12 DAG (Fig. 2C). Plants grown with an LB Pi supply were the only ones to show an increase in root to shoot ratio. Plants grown with an MB Pi supply had the lowest root to shoot ratio, indicating the most efficient growth. In contrast, plants grown with unbuffered Pi show no significant differences in their root to shoot ratios, regardless of the Pi concentration. At 9 DAG, plants grown on LB and LU media had similar fresh weights and Pi concentrations; however, plants grown on LB media amassed a greater total biomass at 12 DAG and shifted a substantial portion of their overall biomass to root growth rather than shoot growth.

### Root architecture is altered by Pi supply

**Fig. 3.**
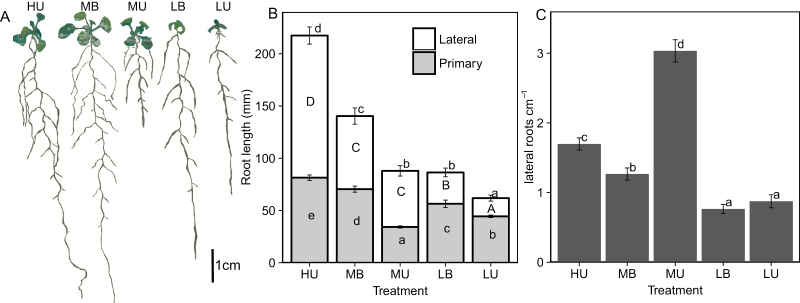
Root growth and root architecture of plants grown on different media. (A) Representative plant images from each medium. (B) Root lengths of primary (white) and lateral (gray) roots for plants from each treatment. Bars represent the average (*n*=60) with the SE for primary root length (lower) and total root length (upper). Significance determined via Tukey’s HSD (*P*<0.05) within groups [primary (lower case, in white bars), lateral (upper case, in gray area), and total (lower case, above)] root length. (C) Lateral root density was determined using the same plants as for total architecture.

In the MU treatment, primary root length was significantly decreased, and root branching increased, especially when considered as root branching density, since the primary root axis was much shorter (Fig. 3). Buffered plants, both MB and LB, had greater total root length and greater primary root length than their unbuffered counterparts (Fig. 3). Plants grown on LB media invested more energy into increasing axial growth and decreasing branching, a markedly different phenotype from that manifested by plants grown on unbuffered media.

**Fig. 4.**
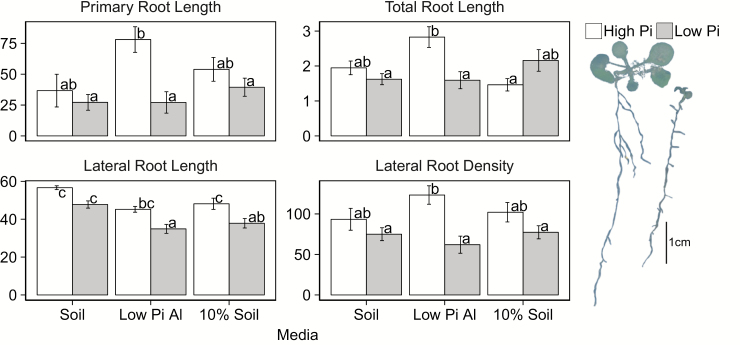
Root architecture of soil-grown plants. Parameters were determined from scans (*n*=6) of plants harvested 12 DAG from each growth condition. Sample plants (right) were harvested from the low-Pi Al_2_O_3_ medium mix supplemented with high- (left) or low- (right) Pi fertilizer.

Since plants grown on gel media are often used as a model system to simulate plant growth and behavior in soil, we grew Arabidopsis plants in soil to compare root architecture in Pi-limiting soil conditions with that observed on agar plates. Plants were grown in each of three media mixes: (i) low-Pi field soil; (ii) sand:vermiculite:low-Pi buffered alumina [60:39:1 (v/v/v)]; or (iii) sand:vermiculite:field soil [55:35:10 (v/v/v)]. Root system architecture in these media was more variable than that of gel media-grown plants, probably due to the much more heterogeneous environment of soil as compared with a gel plate system (Fig. 4). Plants were smaller in soil at 12 DAG, resembling the size of plate-grown plants at 9 DAG. An increase in lateral root branching analogous to that seen in the MU plates was not seen in any of the soil-grown plants. Under low-Pi conditions, all plants were smaller, with shorter primary roots, less lateral root length, and less total root length (Fig. 4). Lateral root branching density was either significantly less under low Pi conditions or was not impacted. In these conditions, where P is buffered by soil or alumina, root growth is similar to that in the buffered gel growth system.

### Root hairs respond to low Pi differently in buffered and unbuffered systems

**Fig. 5.**
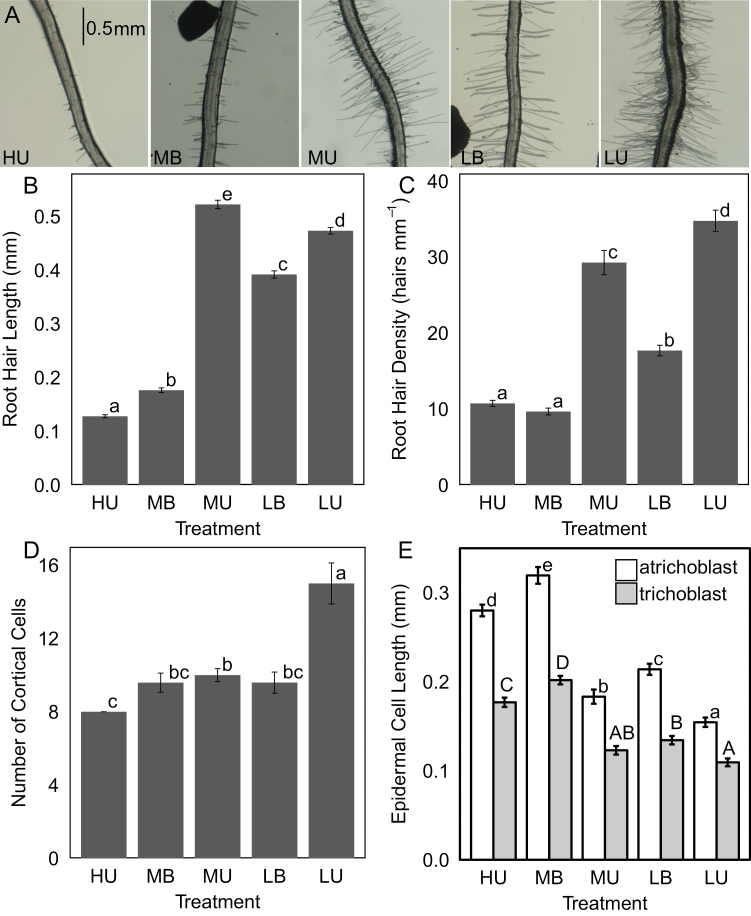
Root hair growth of plants on different media. (A) Sample images of root hairs from each treatment. For all, bars represent the average with SE, and significance determined by Tukey’s HSD (*P*<0.05) (B) Root hair length determined by measuring five root hairs from each of 60 plants. (C) Root hair density determined by counting the number of root hairs in a segment of known length on 60 plants per treatment. (D) Number of cortical cells per plant (*n*=10). (E) Lengths of trichoblasts and atrichoblasts of the epidermis. Five cells of each type were measured per plant (*n*=20). Letters indicate differences determined by Tukey’s HSD (*P*<0.05) within each cell classification; atrichoblasts have lower case letters, trichoblasts, upper case.

Plants are known to increase the length and density of their root hairs under Pi-limiting conditions. Root hairs of plants grown on LB media had increased root hair length and density as compared with those grown on MB media, but the increase was not as dramatic as the 4-fold increase seen in MU compared with HU (Fig. 5). Root hairs of plants grown on unbuffered media were significantly denser as compared with their buffered counterparts.

We examined two factors that contribute to the increase in RHD: epidermal cell length and cortical cell number (Ma *et al.*, 2001). Plants grown on unbuffered media had significantly more cortical cells with decreasing P concentration as compared with the consistent eight cells observed in HU plants (Fig. 5). Plants grown on buffered media had an average of 9.59 cells for plants grown on both MB and LB media, with counts ranging from 8 to 14 cortical cells per root. Treatments that resulted in increased RHD also had decreased epidermal cell length, and epidermal cell length differed between trichoblasts and atrichoblasts as expected. The ratio of atrichoblast to trichoblast length ranged from 1.44 for plants grown on LU media to 1.62 for plants grown on HU media, but there were no significant differences among treatments (not shown).

### Gene expression of common Pi stress genes differs in buffered and unbuffered Pi-grown plants

**Fig. 6.**
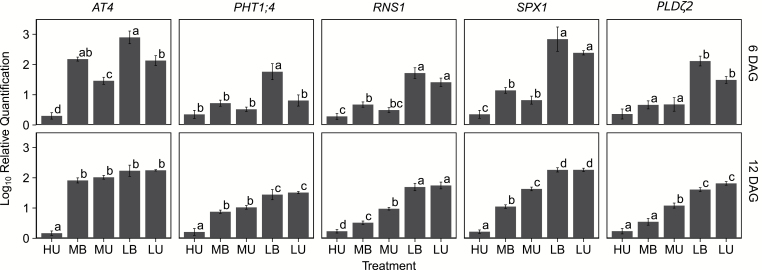
Relative expression of five canonical Pi stress response genes in roots of plants grown on different media. Expression was measured at both 6 DAG (top row) and 12 DAG (bottom row) DAG. Bars represent averages of three different replicates, with 20 plants per replicate. Letters indicate significance determined by Tukey’s HSD (*P*<0.05) for each gene and each day.

Though the expression of hundreds of genes is regulated by P stress (O’Rourke *et al.*, 2013; Li and Lan, 2015; Sun *et al.*, 2016), certain genes have been used as markers in many different studies. We profiled the expression of some of these canonical genes at both 6 and 12 DAG (Fig. 6). At 6 DAG, plants were not yet displaying dramatic symptoms of P stress. At this time, *AT4*, a non-coding transcript whose expression is strongly induced in roots under low P (Shin *et al.*, 2006), was already differentially expressed among treatments; LB plants had the highest relative expression and HU plants the lowest. By 12 DAG, the expression was more uniform across treatments, and only HU plants had significantly lower expression levels than other treatments. The inorganic Pi transporter 1;4 (*PHT1;4*), which is known to be up-regulated in roots under Pi starvation (Shin *et al.*, 2004), shows increased expression in LB-grown plants at 9 DAG, indicating that LB plants are experiencing an earlier stress than their unbuffered counterparts. The expression of *PHT1;4* is Pi concentration dependent in both buffered and unbuffered systems at 12 DAG.

Expression profiles of three additional genes, phospholipase D zeta-2 (*PLDζ2*), ribonuclease 1 (*RNS1*), and *SPX1*, further indicate the importance of timing in defining the low-Pi response. *RNS1* expression increases rapidly in all plants grown on low-Pi media. A delayed increase in expression occurs in MU plants, while MB plants have a consistent, slightly increased expression. *SPX1*, encoding a nuclear protein that has increased expression under Pi stress and is known to interact with PHOSPHATE STARVATION REPONSE1 (*PHR1*) (Puga *et al.*, 2014), has a similar expression profile to *RNS1*. At 12 DAG, MU plants have a slightly higher expression of *SPX1* than their buffered counterparts. Expression of all of these genes is lowest in HU plants.

### Iron interacts with Pi to shape the phenotype

**Fig. 7.**
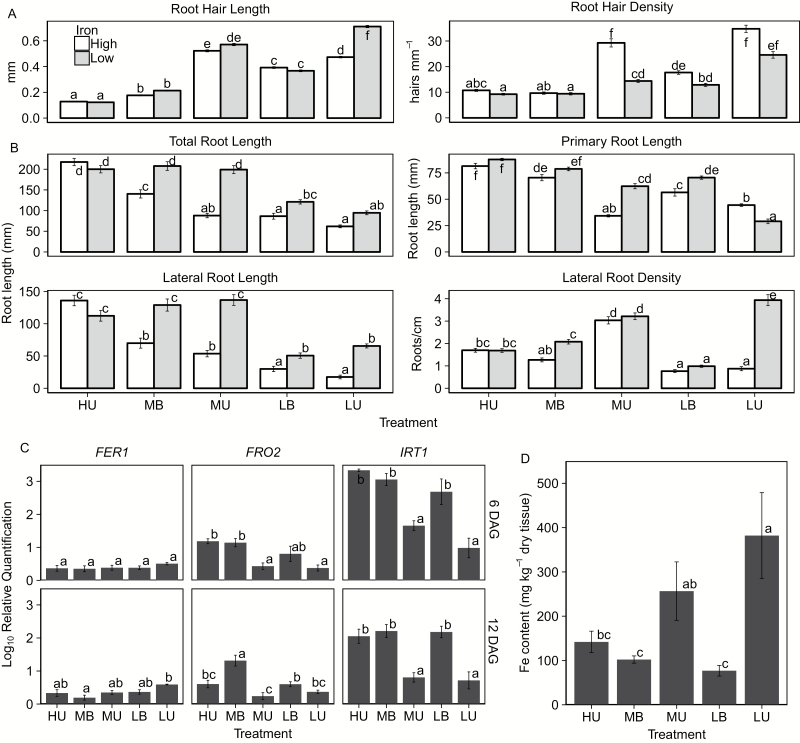
Impact of reduced Fe in gel in combination with Pi delivery on plant growth. High Fe is standard medium (50 μM) and low is 2.5 μM. (A) Root hair length and density measured as previously described (*n*=30 plants per treatment). (B) Root architecture measurements (*n*=30 plants per treatment). (C) Gene expression of genes known to be modulated by both Pi and Fe supply (*n*=20). All plants were grown under high Fe (50 μM). (D) Shoot Fe content determined via ICP-MS (*n*=5) from plants grown under high Fe (50 μM).

The low-Pi phenotype of Arabidopsis is known to be partially shaped by the presence of excess available Fe in the growth medium (Ward *et al.*, 2008). To examine the role of Fe in buffered media, we grew plants on media containing 2.5 µM Fe (as Fe-DTPA), versus 50 µM Fe in all other media in this study. Reducing the Fe concentration of the media affected the phenotype in all treatments, as expected since Fe is a known growth regulator (Broadley *et al.*, 2012). Decreased Fe concentration resulted in significantly longer primary roots under MU and LB conditions (Fig. 7). Under LU conditions, the primary root was significantly shorter, and the plants had increased branching. Under LU, low-Fe conditions, root hair length increased significantly, a known growth response to low Fe (Schmidt *et al.*, 2000). Root hair density decreased in MU-grown plants, probably due to the improved growth of the primary root.

To explore these nutrient interactions further, we analyzed the expression of three genes related to Fe and P, *IRT1*, *FER1*, and *FRO2*, while also measuring plant Fe content (Fig. 7). Previous work has shown that *FER1* expression increases under low-Pi conditions, whereas *IRT1* and *FER1* have decreased expression under Pi stress (Ward *et al.*, 2008; Bournier *et al.*, 2013; Rai *et al.*, 2015). *FER1* expression did not differ among treatments at 6 DAG, and at 12 DAG only MB plants had significantly lower expression than LU plants. *FRO2* expression was significantly lower under MU and LU conditions at 6 DAG. At 12 DAG, MB plants had significantly higher expression of *FRO2* as compared with all unbuffered treatments. Expression of *IRT1* was significantly lower in MU and LU conditions compared with HU, MB, and LB conditions at 6 and 12 DAG. Plant Fe concentration measurements show that, unlike their buffered counterparts, MB- and LB-grown plants did not overaccumulate Fe.

## Discussion

P is often limiting to plant growth and crop productivity, but most of our understanding of the molecular responses to P stress is drawn from the highly artificial Arabidopsis gel media growth system. Here, we present a modification to the gel-based system that is simple, reproducible, and allows for direct observation of plant growth. By adsorbing Pi on commercially available Al_2_O_3_ particles, we are able to deliver buffered Pi regimes to Arabidopsis that mimic P regimes in natural soil. By making slight modifications to the widely used plate-based system, we maintain the advantages of a plate-based system while providing plants with realistic Pi regimes. We also limit the complications that arise due to nutrient toxicities when Pi is eliminated.

In soil, P is exchanged between soil particles and the soluble fraction in equilibrium reactions (Holford, 1997; Shen *et al.*, 2011). Plant growth and P assimilation remove P from the soluble fraction where P flows via diffusion, leading to the release of Pi from bound pools into the soluble pool to maintain equilibrium. The dynamic nature of this equilibrium allows plants to accumulate much higher concentrations of P than what is available in the soil at any one point in time (Holford, 1997; Raghothama, 1999; Heuer *et al.*, 2017). This is in contrast to the classic gel system in which freely available Pi is present in millimolar concentrations, far exceeding the concentrations in fertile soil. Plants grown with this unrealistic level of Pi are used as the controls with which all other treatments are compared. We have shown that plants grown with a much lower concentration of Pi (medium buffered, MB) that is delivered in buffered form grow just as well as those under traditional, unbuffered, high-Pi (HU), conditions. Plants grown under sufficient, buffered Pi conditions have different levels of expression of canonical Pi stress genes, including *AT4*, *PHT1;4*, *RNS1*, and *SPX1*, than plants grown under high-Pi, unbuffered conditions. Since the high-Pi, unbuffered condition is highly artificial, many genes previously identified as Pi responsive may not be responsive to differences in Pi levels under buffered conditions. Here, we have shown that *AT4*, a gene commonly used as an indicator of Pi stress (Shin *et al.*, 2006), has greater expression under sufficient, buffered Pi conditions as compared with high, unbuffered conditions (Fig. 6), despite the fact that plants grown under sufficient levels of buffered Pi are not experiencing obvious Pi stress. Three other genes, *PHT1;4*, *RNS1*, and *SPX1*, that play roles in uptake of Pi (Shin *et al.*, 2004), sensing and responding to Pi stress (Bariola *et al.*, 1994), and early Pi-deficient signaling (Puga *et al.*, 2014), respectively, also show significant expression differences between high, unbuffered and medium, buffered treatments at either 6 DAG (*RNS1* and *SPX1*) or 12 DAG (all three genes). Of the genes we analyzed for expression, only *PLDζ2*, which plays a role in remobilizing phosphate from internal pools (Cruz-Ramírez *et al.*, 2006), did not differ between HU and MB plants, though expression was significantly greater in buffered and unbuffered low-Pi conditions at 6 and 12 DAG, and in medium, unbuffered conditions at 12 DAG, indicating that it is Pi stress responsive. All the genes we analyzed showed some degree of Pi deficiency response, but the magnitude and timing of this response differed under buffered versus unbuffered conditions. Two previous studies have profiled expression of P stress-related genes in soil-grown plants, both examining proton-coupled Pi transporters. Though increases in expression were found under Pi stress, they were not profiled in a time-dependent manner, probably due to the difficulty of retrieving roots from soil (Mudge *et al.*, 2002; Rausch *et al.*, 2004). We recommend that the expression and function of many of the genes thought to be involved in the Pi stress response be re-examined under buffered conditions, which would be easier to work with than soil. This will not only further our understanding of the Arabidopsis Pi deficiency response, but will also strengthen the possibility of discovering conserved responses that could have impacts in crops and soil-grown plants.

The classic gel media system has come under scrutiny previously, as have similar hydroponic methods. The gelling agents used (Jain *et al.*, 2009; Gruber *et al.*, 2013), pH of the media (Svistoonoff *et al.*, 2007), exposure of roots to light (Xu *et al.*, 2013; Rellán-Álvarez *et al.*, 2015), and nutrient composition (Strieder *et al.*, 2017) have all been shown to affect plant responses to nutrient stress. The complex interactions between micro- and macronutrients in media have been described (Misson *et al.*, 2005; Gruber *et al.*, 2013; Kellermeier *et al.*, 2014). Recently, Strieder *et al.* (2017) grew plants on modified, Pi-limited Somerville and Ogren (SO) and half-strength Murashige and Skoog (MS) media and found that the *phosphate deficiency response9* (*pdr9*) mutant of Arabidopsis is not actually a Pi-sensitive mutant as previously reported (Chen *et al.*, 2000), but rather is hypersensitive to ammonium and Fe. Though using alternative media may eliminate certain confounding nutrient conditions, the Pi is still unbuffered. Rescreening of mutant lines that have been identified as Pi sensitive or tolerant to low Pi should be done using a buffered Pi system, rather than any media where the Pi is not buffered. Doing so will probably yield results that may require reconsideration of the nature of P stress responses in Arabidopsis and what genes and pathways shape this behavior.

Genes involved in the maintenance or control of primary root elongation under low-Pi conditions have been identified by examining the elongation of mutants under various Pi conditions (Chen *et al.*, 2000; Sánchez-Calderón *et al.*, 2006; Svistoonoff *et al.*, 2007; Ticconi *et al.*, 2009; Wang *et al.*, 2010*a*; Strieder *et al.*, 2017). Here, we show that although primary root length decreased under low, buffered conditions, the decrease was not as drastic as what is observed under unbuffered conditions. When grown with buffered Pi, plants decreased their branching density under low Pi conditions as compared with sufficient (medium) Pi conditions, in contrast to the response observed in unbuffered, medium versus unbuffered, high-Pi-grown plants (Fig. 3). Since the root architecture of plants grown on gel with a buffered Pi supply more closely resembles that of plants recovered from a soil or sand culture system (Fig. 4), investigations of genetic components playing a role in more realistic Pi stress responses are more likely to have implications for plants grown in agricultural conditions. The reduction in branching and promotion of axial growth observed in buffered Pi is similar to what has been observed in maize, common bean, and rice in greenhouse and field studies (Borch *et al.*, 1999; Mollier and Pellerin, 1999; Vejchasarn *et al.*, 2016). As described in many other species (Gahoonia and Nielsen, 1997; Zhu *et al.*, 2010; Miguel *et al.*, 2015; Wang *et al.*, 2016; Vejchasarn *et al.*, 2016), root hair length and density increase under low-Pi conditions in Arabidopsis under both buffered and unbuffered growth conditions. Previously, we had shown that the increase in RHD under Pi-limiting conditions was partially due to an increase in cortical cell number, and thus in the number of trichoblast files forming root hairs (Ma *et al.*, 2001). Here we show that under conditions where Pi is delivered through a buffered regime, cortical cell number does not increase. The increase in RHD is instead due to a decrease in epidermal cell length, resulting in more cells per area measured.

The response of Arabidopsis to low P is known to affect micronutrient nutrition, including copper (Cu) and Zn (Misson *et al.*, 2005; Perea-García *et al.*, 2013; Khan *et al.*, 2014; Briat *et al.*, 2015), and, most notably, Fe. The commonly described phenotypic response to low Pi that results in the arrest of the primary root can, in many cases, be ascribed to excess Fe availability in the absence of P (Schmidt and Schikora, 2001; Ward *et al.*, 2008; Lan *et al.*, 2012; Bournier *et al.*, 2013; Li and Lan, 2015; Sun *et al.*, 2016). Recent work claims that 579 genes are co-regulated by P and Fe (Li and Lan, 2015), but these experiments were conducted using a traditional, unbuffered system; therefore, many of these genes may not actually be Pi responsive under buffered Pi conditions. Our system eliminates confounding Fe–Pi issues, since our plants experiencing Pi stress are not overaccumulating Fe, nor do they respond to Fe stress at the transcriptional level at low-Pi conditions. Adding Al_2_O_3_ particles to the media can also alter other nutrients, including B, Mo, and Zn. To address this, we adjusted the concentrations of these elements in our nutrient solution. These adjustments lead to similar availability (B) or plant accumulation (Zn) of these nutrients (Supplementary Fig. S1). Mo concentrations in plant tissue differed between buffered and unbuffered treatments; however, the activity of nitrate reductase, for which Mo is required, did not differ between treatments, indicating that these differences were unlikely to be functionally relevant. The buffered delivery of Pi not only creates realistic P regimes, but also eliminates known problems that arise when P is eliminated from nutrient solutions. The use of buffered Pi regimes should not be restricted to studies focusing on Pi nutrition. Salinity stress has long been known to interact with Pi stress, for example in soybean (Grattan and Maas, 1984; Phang *et al.*, 2009) and barley (Talbi Zribi *et al.*, 2011). Recently, salinity and Pi stress have been shown to interact to shape root system architecture in a diverse collection of Arabidopsis ecotypes (Kawa *et al.*, 2016). It is unknown whether any of the identified responses are due to the artificiality of Pi regimes or are responses that would be observed if plants were grown in a buffered Pi or soil system.

The standard growth method for Arabidopsis has allowed for rapid, uniform growth that has led to countless conclusions in plant biology. The simplification of the medium has led to the elimination of diffusion limitation caused by soil chemistry, resulting in responses that may not be relevant to those of plants grown in soil. In addition to complications from excess Fe, plants grown in gel without buffered P may be responding to the perceived decline in P availability, complicating interpretation of responses. Our buffered system more closely mimics soil and results in a more realistic and logical growth phenotype. Phosphate adsorbed onto Al_2_O_3_ particles (Al-P) can easily be made in any lab using standard equipment in <1 d. By continuing to use a gel-based system, advanced imaging, including confocal microscopy and kinematics, can still be conducted without the need for expensive systems such as MRI or CT scanning. We recommend that all studies of mineral nutrition in a gel system, especially those pertaining to Pi nutrition, adopt this buffered system for future studies.

## Supplementary data

Supplementary data are available at *JXB* online.

**Fig. S1**. Micronutrient availability in gel and plant micronutrient content.

**Table S1**. Primers used in this study for qRT-PCR.

Supplementary Figure S1 and Table S1
