# Supplementary material for: Buffered delivery of phosphate to Arabidopsis alters responses to low phosphate
Source: J Exp Bot. 2018 Jan 3;69(5):1207–19. doi: 10.1093/jxb/erx454 (PMC6019003; doi:10.1093/jxb/erx454)
Supplement: Supplementary Figure S1 and Table S1 [file erx454_suppl_supplementary_figure_s1_and_table_s1.pdf]

Supplemental Materials and Methods

Nitrate reductase assay was determined following published methods (Cheeseman and Tankou, 2005). Protein concentration was determined via a Bradford assay (B6916 Sigma-Aldrich) using BSA as a standard.

Gel nutrient analysis was completed following drying (60°C for four days) and ashing (470°F for 8 hours) and digestion in 0.1N HCl. Digests were analyzed on an Agilent 730-ES, axial torch orientation (Agilent Technologies, Santa Clara, CA) following EPA method 6010.

Plant nutrient analysis was completed following drying (60°C for four days) and digestion in concentrated, ultra-pure nitric acid (HNO_3) for 8 hours at 80°C in teflon vessels. The solution was diluted to obtain a final HNO_3 of 0.45 N. Samples were run on a Thermo Fisher Scientific XSeries 2 both with and without collision cell technology as required (srm 1640a, NIST 1547).

Table S1: Primers used in this study for qRT-PCR.

Gene	Forward	Reverse	TAIR gene model
Actin7	GCCATTCAGGCCGTTCTTT	ATCGAGCACAATACCGGTTGT	At5g09810
IRT1	CGTTTCGTTCCTCCAACCA	CGGAGGCGAAACACTTAATGA	At4g19690
Pht1;4	GTGCCGGCCGAAATCTT	AGAGATACCGTGGCAGGTTGA	At2g38940
PLDZ2	TGGTGGTTATGTCCGGAGCTA	AGCCGCAATGATGGATGATC	At3g05630
RNS1	CTTGCCCAAGCGGTTCAG	GTACCATGCTTCTCCCATTCG	At2g02990
SPX1	TGCCTTGCGGGTTTTGAA	CGGCAATGAAAACACACTAACAGT	At5g20150
AT4	GCCATCCCCTAAAGAAACTGAA	ACCCTTTATTGGTGAGATCTTCTGA	At5g03545
FER1	CTTTCACCGCCGCTAATCC	GAGAAACCGACGGAGAAGCA	At5g01600
FRO2	GTCTTGCCATTATCCGGTCTTG	TCGGGTTATGAAGGCTTCGA	At1g01580

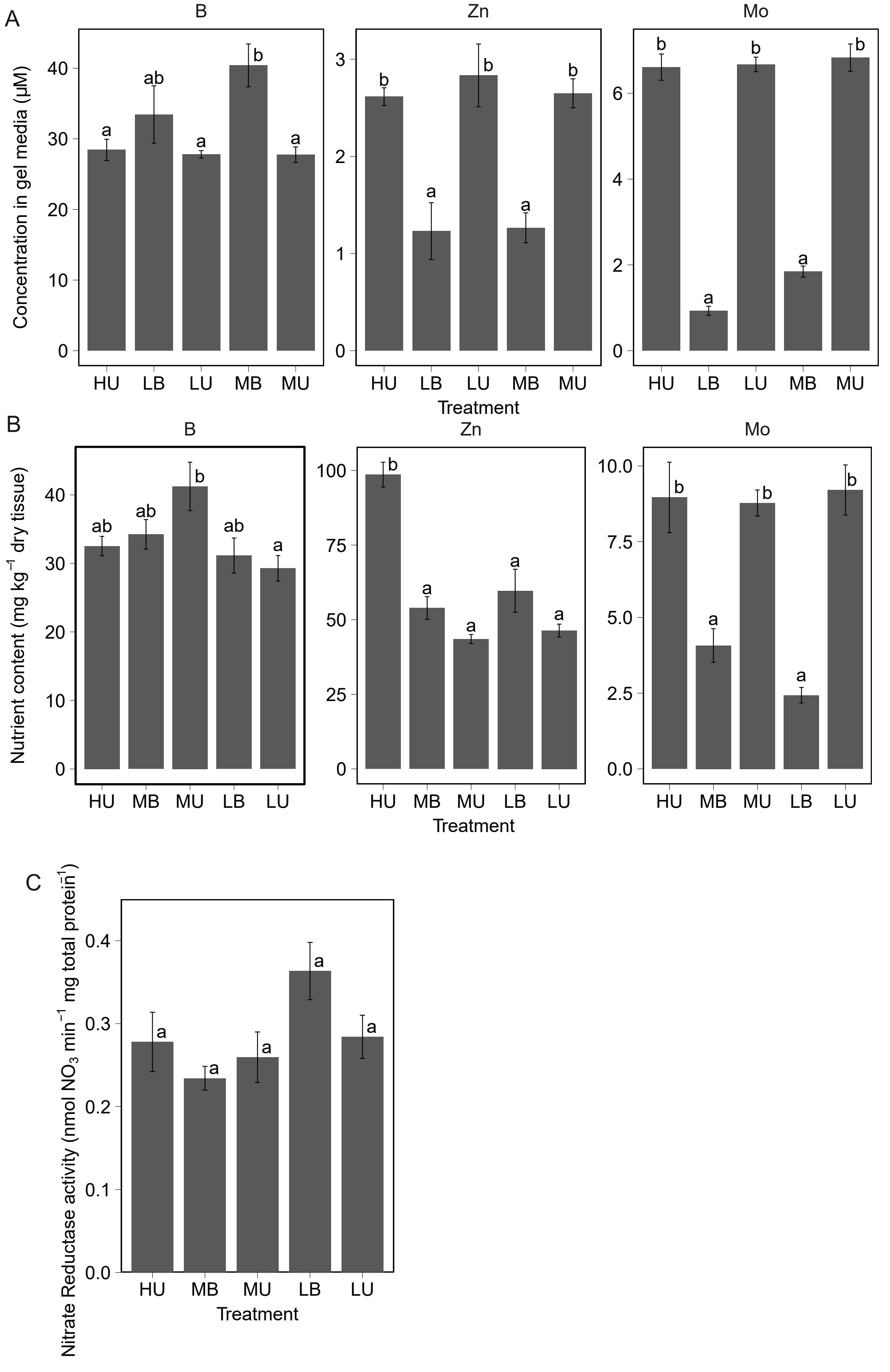

Figure S1: Micronutrient availability and plant content. A) Available B, Zn, and Mo in the gel media of each treatment. All levels were adjusted as described in Materials and Methods. Content was determined via ICP-AES of media without plants (n = 5). B) Micronutrient content of shoots determined via ICP-MS (n = 5). C) Nitrate reductase activity was measured to determine the presence of sufficient Mo (n = 5).
